# Inflammaging increases susceptibility to cigarette smoke-induced COPD

**DOI:** 10.18632/oncotarget.4027

**Published:** 2015-05-24

**Authors:** Gerrit John-Schuster, Stefanie Günter, Katrin Hager, Thomas M. Conlon, Oliver Eickelberg, Ali Önder Yildirim

**Affiliations:** ^1^ Comprehensive Pneumology Center, Institute of Lung Biology and Disease, Helmholtz Zentrum München, Member of the German Center for Lung Research (DZL), 85764 Neuherberg, Germany; ^2^ Klinikum der Universität München, 81377 München, Germany

**Keywords:** aging, B cells, iBALT, COPD, cigarette smoke, Gerotarget

## Abstract

Chronic obstructive pulmonary disease (COPD) is related to an abnormal chronic inflammatory response of the lung to mainly cigarette smoke (CS) and the disease risk is increased in aged individuals. The source of this chronic inflammation is due to the repeated and progressive activation of immune cells. We hypothesize that in a chronic CS-induced mouse model, the predisposition to COPD pathogenesis in aged mice is characterized by an elevated immune response compared to young animals. We measured several characteristics of COPD in young and old mice (2 and 12 months of age) exposed to CS for 3 months. CS-exposed aged mice exhibited increased lung compliance (0.061 ± 0.008 vs. 0.055 ± 0.006 ml/cm H_2_O, *p* < 0.01), emphysema development (35.36 ± 0.71 vs. 25.31 ± 0.005 μm; *p* < 0.01) and airway remodeling (2.15 ± 0.37 vs. 1.09 ± 0.64 μm^3^/μm^2^; *p* < 0.01) compared to control animals, which was not seen in CS-exposed young mice. Quantification of lung tissue inflammation revealed a significantly greater volume of inducible bronchus-associated lymphoid tissue structures in aged mice after CS exposure (5.94 ± 2.89 vs. 2.37 ± 1.69 μm^3^/μm^2^; *p* < 0.01). Our results indicate that age-induced lung inflammation is further elevated after CS exposure in old mice, potentially via an age-induced change in immune cell susceptibility to CS thereby accelerating the pathophysiological hallmarks of COPD.

## INTRODUCTION

Aging is a natural process characterized and influenced by mechanisms such as increased oxidative stress, telomere shortening, and cellular and immunosenescence (a state of permanent growth arrest) [1]. The resulting anatomic and physiological changes affect both functional organ properties and the ability to mount appropriate responses to infections and injury, thereby leading to an increased incidence of a variety of chronic diseases in the elderly including respiratory diseases such as chronic obstructive pulmonary disease (COPD) [2].

The pathogenesis of COPD is characterized by an abnormal lung inflammatory response to chronic exposure to toxic gases and particles, most often cigarette smoke (CS), which induces severe pathophysiological changes in the lung [3]. These include mucus production, remodeling of small airways, chronic bronchitis and septal tissue damage leading to the constant and accelerated decline in lung function observed in patients suffering from COPD. The source of this chronic inflammation of the lung is mainly attributed to the progressive activation of immune cells over time. Chronic CS exposure initiates a complex inflammatory cascade characterized by activation and influx of various inflammatory cells and secretion of inflammatory mediators into the lung [4]. The inflammatory cell response includes both innate, predominantly macrophages and neutrophils [5], and adaptive immune cells, specifically T and B lymphocytes [6]. Their numbers are increased in the lungs of patients with COPD [7]. In both young and old mice, six months long term CS exposure induced enhanced inflammation with similar pathophysiological responses, in particular emphysema and small airway remodeling [8]. However, this study did not address potential differences in the early response to CS, which may account for the limited number of smokers developing COPD.

Age-related changes of immune system functions have been described, but are complex phenomena incompletely understood [1]. Immunosenescence of both innate and adaptive immune cells not only leads to a reduction in diversity and functionality of certain cell types, but also to clonal expansion and activity of others [9]. The consequence is a chronic low-grade inflammation associated with aging (inflammaging) and age-related diseases, which is also observed for the lung [10].

The age-related systemic and/or local alterations of the immune system associated with a chronic inflammatory condition and a possible lack of regulation might also play an important role in COPD development and progression [9]. The changes that occur in COPD often mimic changes seen in the aging lung, and these processes are suggested to accelerate lung aging in COPD patients [11]. For example, in severe emphysema patients, CS-induced lung inflammation was amplified compared to healthy smokers [12]. In a mouse model of short-term CS exposure, aging enhanced the susceptibility to early CS-induced inflammation through baseline increases in chemokine expression by bronchial epithelial cells [13]. Furthermore, 8 weeks CS exposure of mice deficient in anti-senescence protein senescence marker protein-(SMP)30 [14], and 6 months CS exposure of telomerase RNA-null (*mTR^−/−^*) mice promoted airspace enlargement [15], pointing to a contribution of immunoaging to accelerated COPD pathogenesis at earlier disease development stages.

Recently, we described pathophysiological changes in young mice (2 months of age) in a model of COPD starting from 4 months of CS exposure [6]. In order to distinguish a possible increase in susceptibility to COPD development in the context of aging, we hypothesized that after less than 4 months of CS exposure, the pathogenesis of COPD will be characterized by an elevated immune response in aged compared to young mice. We measured several pathophysiological hallmarks of COPD in these animals after 3 months of CS exposure and found that lung inflammation (presence of innate and adaptive immune cells, inducible bronchus-associated lymphoid tissue formation and cytokine expression) after CS exposure was accelerated in aged mice. This led to increased lung compliance, emphysema development and small airway remodeling in aged animals, whereas young mice did not show any development of COPD characteristics at this early stage. These findings suggest a role for age-related inflammatory changes of immune cells involved in accelerated COPD pathogenesis.

## RESULTS

### Aged mice are prone to develop emphysema and airway remodeling after CS exposure

**Figure 1:**
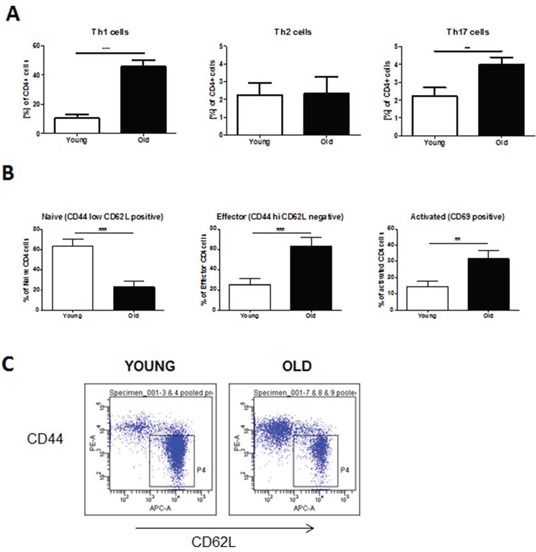
Baseline levels of CD4+ T cells are increased in lungs and spleens of aged mice For flow cytometric analysis single-cell suspensions were prepared from whole lung tissue or spleens of young (2 months of age) and old (12 months of age) mice. Surface marker and intracellular cytokine stainings were subsequently performed. **A.** Th1 cells were defined by expression of CD4 and IFNγ, Th2 cells by expression of CD4 and IL-4, and Th17 cells by expression of CD4 and IL-17A. **B.** CD4^+^ effector and activated T cells were characterized by expression of CD44 and CD69, respectively; **C.** Representative FACS plots of stainings for CD4^+^ effector T cells. Data were combined from 2 independent experiments with *n* = 4 and are given as mean values ± SD; one-way ANOVA following Bonferroni post test with *p < 0.05, **p < 0.01, ***p < 0.001.

To investigate whether aged mice exhibit different levels of inflammatory cells at baseline, we stained single-cell suspensions of non-exposed lungs and spleens from young (2 months) and old (12 months) animals for Th cell subsets. We observed a significant increase in baseline levels of Th1 and Th17 cells in the lungs of aged compared to young mice (Fig. 1A). Moreover, the spleens of aged mice also contained significantly higher numbers of both CD4^+^ effector T cells as determined by staining of CD4^+^ cells for CD44 and CD62L and CD69^+^ early activated CD4^+^ T cells (Fig. 1B & 1C).

**Figure 2:**
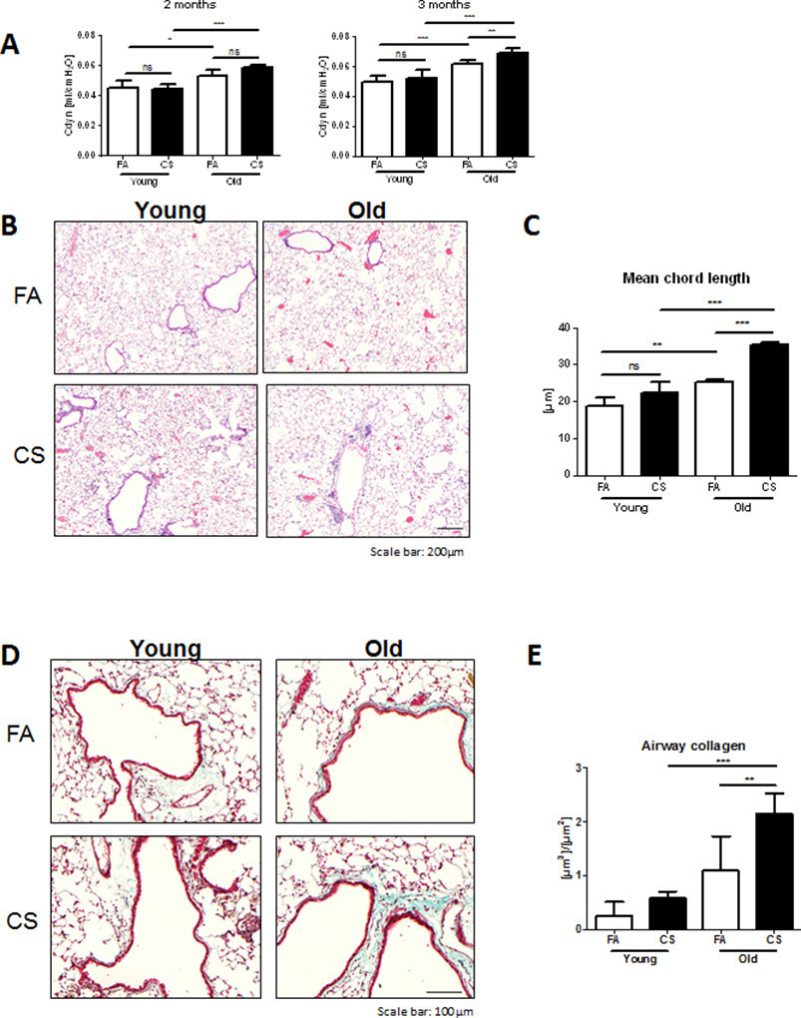
Aged mice develop emphysema and airway remodeling after 3 months of CS exposure **A.** Lung function measurements for dynamic lung compliance were performed in intubated animals after 2 months of CS exposure and in tracheostomized animals after 3 months of CS exposure. **B.** Representative micrographs of HE-stained lung tissue sections from young and old FA and CS-exposed mice; scale bar 200 μm. **C.** Quantitative measurement of emphysema was determined by design-based stereology of HE-stained lung tissue sections using an Olympus BX51 light microscope equipped with the computer-assisted stereological toolbox newCAST. **D.** Representative micrographs of Masson's Trichrome staining of lung tissue sections from young and old FA and CS-exposed mice; scale bar 100 μm. **E.** Total volume of airway collagen per basal membrane was determined via quantitative morphological assessment. Data were combined from 2 independent experiments with *n* = 8 and are given as mean values ± SD; one-way ANOVA following Bonferroni post test with **p < 0.01, ***p < 0.001.

Therefore, we investigated whether age-related inflammation could lead to earlier development of pathophysiological hallmarks of COPD in aged mice after CS exposure. To this end, young and old mice were exposed to CS for 2 months. Lung function analysis of mice after 2 months of CS exposure revealed only age-dependent differences for dynamic lung compliance, as indicated by significant increases for aged control and CS-exposed compared to young mice (Fig. 2A). For this reason, we exposed the same animals to CS for another month. Interestingly, after 3 months of CS exposure, aged mice showed significantly increased values for dynamic lung compliance compared to CS-exposed young animals. This was confirmed by histological analysis of HE-stained lung tissue sections (Fig. 2B). Morphometric quantification of emphysema development also indicated a significantly increased mean chord length in aged control versus young animals (Fig. 2C). Interestingly, CS exposure led to further increases in mean chord length in aged mice, whereas young mice failed to develop emphysema after 3 months of CS exposure.

CS-induced remodeling of airways is one of the hallmarks observed in COPD patients [3]. Therefore, we investigated whether CS exposure caused increased collagen deposition around the airways in our animals. Masson's Trichrome staining of lung tissue sections revealed higher collagen deposition around airways after CS exposure in aged compared to young mice (Fig. 2D). FA control animals showed positive staining mostly around vessels; however, aged mice already had slight baseline increases in collagen deposition around the airways. These findings were confirmed by morphometric quantification of airway collagen deposition (Fig. 2E), with a trend towards higher levels in aged compared to young control animals and significantly increased values after CS exposure in aged mice.

These results suggest that the increased baseline inflammation in aged mice led to earlier development of the hallmark features of COPD after CS exposure, specifically emphysema development and airway remodeling.

### Aged mice exhibit higher lung tissue inflammation associated with iBALT formation after CS exposure

**Figure 3:**
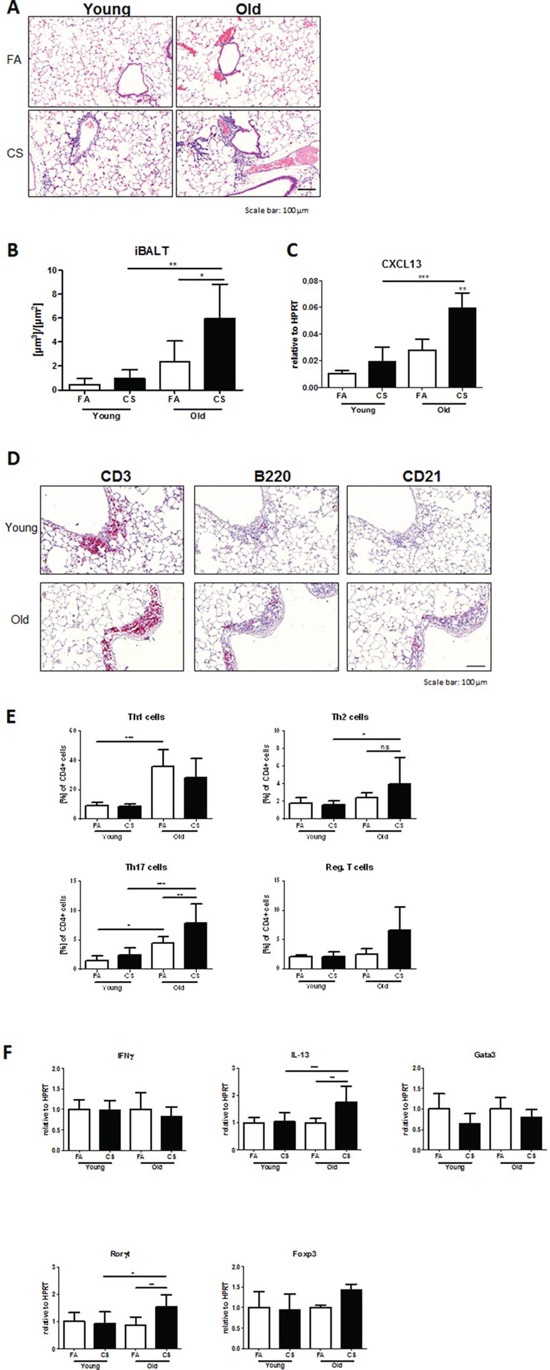
Development of iBALT is increased in aged CS-exposed mice and consists of T and B lymphocytes and follicular dendritic cells **A.** Representative micrographs of HE-stained lung tissue sections from young and old FA and CS-exposed mice showing the presence of iBALT in aged animals after CS exposure; scale bar 100 μm. **B.** Total volume of iBALT per basal membrane was determined via quantitative morphological assessment. **C.** Lung tissue mRNA expression of CXCL13 was measured by qPCR. **D.** Representative micrographs of immunohistochemical stainings for CD3 (T lymphocytes), B220 (B lymphocytes) and CD21 (FDCs) in lung sections from CS-exposed young and old mice; scale bar 100 μm. **E.** Flow cytometry analysis of whole lung for CD4^+^ T lymphocyte infiltration reveals increases in Th2 and Th17 cells after CS exposure in aged compared to young mice; Th1 cells were defined by expression of CD4 and IFNγ, Th2 cells by expression of CD4 and IL-4, Th17 cells by expression of CD4 and IL-17A, and regulatory T cells by expression of CD4, CD25 and Foxp3. **F.** Lung tissue mRNA expression of Th signature cytokines IFNγ and IL-13 and transcription factors Gata3, Rorγt and Foxp3 was measured by qPCR. Data were combined from 2 independent experiments with *n* = 8 and are given as mean values ± SD; one-way ANOVA following Bonferroni post test with *p < 0.05, **p < 0.01, ***p < 0.001.

We reported recently that lymphoid follicle (LF)-like structures in the lung, termed inducible bronchus-associated lymphoid tissue (iBALT), are involved in emphysema development [6], although little is known about the causative formation of such structures. Histological analysis of lung sections from 3 months CS-exposed young and old mice revealed a strong tissue infiltration with immune cells and subsequent formation of iBALT in CS-exposed aged mice (Fig. 3A). Morphometric quantification of these structures in aged mice showed a significant increase in iBALT volume per basal membrane after CS exposure, while young mice failed to develop iBALT after CS exposure (Fig. 3B). Recently, Litsiou *et al*. discovered that CXCL13 is involved in lymphoid neogenesis in COPD by promoting B cell migration to ectopic sites of lymphoid tissue formation and by upregulating lymphotoxin on B cells, which in turn further induces CXCL13 required for follicle expansion [16]. Analysis of mRNA expression levels of CXCL13 in lung tissue from our model revealed a significant upregulation after CS exposure in aged mice (Fig. 3C).

Accordingly, staining our lung tissue sections for T and B lymphocytes and follicular dendritic cells (FDCs), using antibodies against CD3, B220 (CD45R) and CD21 respectively, revealed strong staining for all markers in areas of iBALT formation and inflammation (Fig. 3D). These results indicate that aged mice are more susceptible to CS-induced lung tissue inflammation and subsequent iBALT formation and confirm a role for CXCL13 in iBALT formation.

As T cells, rather than B cells and FDCs, predominantly accumulated after CS exposure in aged mouse lungs, we further investigated the different CD4^+^ T cell subtypes involved in iBALT formation after CS exposure in aged animals and analyzed single-cell suspensions of homogenized lungs by flow cytometry. This confirmed a baseline increase in Th1 and Th17 cell levels in the lungs of aged compared to young mice (Fig. 3E). After 3 months of CS exposure, an upregulation of Th17 and, more interestingly, of Th2 cells was apparent in aged animals, whereas levels in young mice remained unchanged. Regulatory T cells (Tregs) also showed a trend towards higher numbers in CS-exposed aged mice, while there were no changes for Th1 cells for both young and old animals after CS exposure.

In a next step, we investigated whether transcription factors and cytokines specific for the detected Th responses were also regulated at mRNA level in aged mouse lungs after CS exposure. We observed increases in CS-exposed aged mice for Rorγt, the master transcription factor of Th17 cells, and for IL-13 (Fig. 3F), a Th2-associated cytokine also involved in emphysema development in a transgenic mouse model [17]. However, Foxp3 and Gata3, the transcription factors characteristic for Tregs and Th2 cells, respectively, as well as the Th1-associated cytokine IFNγ were not increased at mRNA level.

These observations suggest that age-related increases in lung tissue inflammation and iBALT formation induced after CS exposure are predominantly associated with elevated levels of Th17 and Th2 cells.

### Increased BAL inflammation after CS exposure in aged mice consists of macrophages, neutrophils and lymphocytes

**Figure 4:**
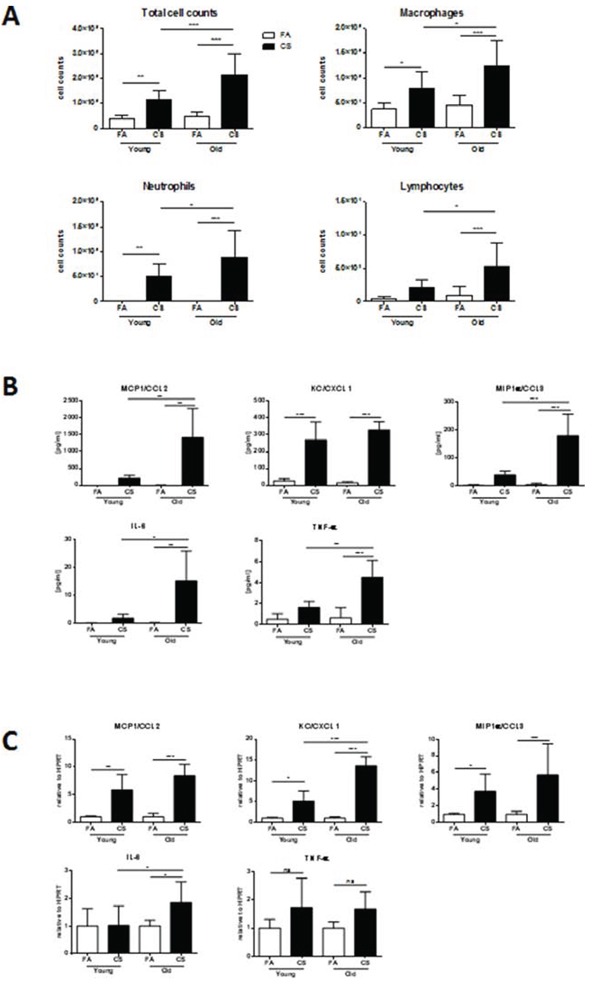
BAL and lung tissue inflammation are increased in aged mice **A.** The lungs of young and aged mice were lavaged with 4 × 0.5 ml aliquots of sterile PBS and total and differential cell counts were determined. **B.** BAL cytokine secretion of MCP1 (CCL2), KC (CXCL1), MIP-1α (CCL3), IL-6 and TNF-α was determined using a magnetic bead-based multiplex assay. For this assay, BAL fluid was concentrated (10x) by ultrafiltration in centrifugal filter devices. **C**. Lung tissue mRNA expression of MCP1 (CCL2), KC (CXCL1), MIP-1α (CCL3), IL-6 and TNF-α was measured by qPCR. Data were combined from 2 independent experiments with *n* = 8 and are given as mean values ± SD; one-way ANOVA following Bonferroni post test with *p < 0.05, **p < 0.01, ***p < 0.001.

Consistent with the observation of higher lung tissue inflammation in CS-exposed aged mice, we detected increased BAL inflammation in these animals as shown by significantly higher total cell counts (Fig. 4A). Elevated BAL cells after CS exposure mainly consisted of macrophages, neutrophils and also lymphocytes, which were significantly increased after CS exposure in aged compared to young mice.

The differences in BAL inflammatory cells were further confirmed on BAL cytokine levels as determined by multiplex assay. The secretion of MCP1, MIP-1α, IL-6 and TNF-α was significantly higher after CS exposure in aged compared to young animals, while KC was similarly upregulated in both groups (Fig. 4B). Interestingly, tissue mRNA expression levels of KC and IL-6 were significantly higher after CS exposure in aged compared to young animals (Fig. 4C). For MCP1 and MIP-1α, a comparable upregulation after CS exposure was observed in both groups.

**Figure 5:**
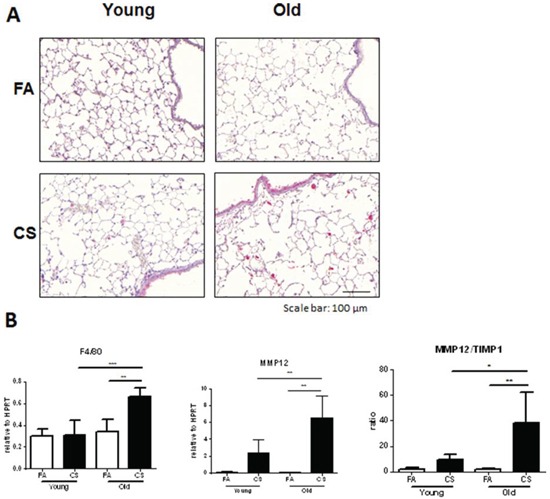
Macrophage markers are increased in the lungs of CS-exposed aged mice **A.** Representative micrographs of immunohistochemical stainings for MMP12 in lung sections from FA control and CS-exposed young and old mice; scale bar 100 μm. **B.** Lung tissue mRNA expression of macrophage markers F4/80 and MMP12 as well as the ratio of MMP12/TIMP1 was measured by qPCR. Data were combined from 2 independent experiments with *n* = 8 and are given as mean values ± SD; one-way ANOVA following Bonferroni post test with *p < 0.05, **p < 0.01, ***p < 0.001.

Because macrophage-derived MMP12 was described as being required for the induction of experimental emphysema after CS exposure [18] and we also observed increased macrophage numbers in BAL fluid of CS-exposed aged mice, we studied the number and localization of macrophages infiltrating the tissue of CS-exposed lungs by performing immunohistochemistry staining for MMP12. We observed significantly higher numbers in CS-exposed aged compared to young mice (Fig. 5A). This was confirmed by determining MMP12 mRNA expression levels, which showed significantly higher CS-induced increases for aged mice (Fig. 5B). Another macrophage marker, F4/80 was also significantly upregulated only in CS-exposed aged animals. Furthermore, the stronger increase in MMP12 expression after CS exposure in aged mice led to a higher MMP12/TIMP1 ratio in these animals, indicating a disturbed balance of MMP12 and its inhibitor TIMP1.

Collectively, these results suggest that the increased level of BAL and lung tissue inflammation and MMP12 upregulation observed in aged mice accelerated COPD development after CS exposure.

### Senescence markers are elevated in aged mice after CS exposure

**Figure 6:**
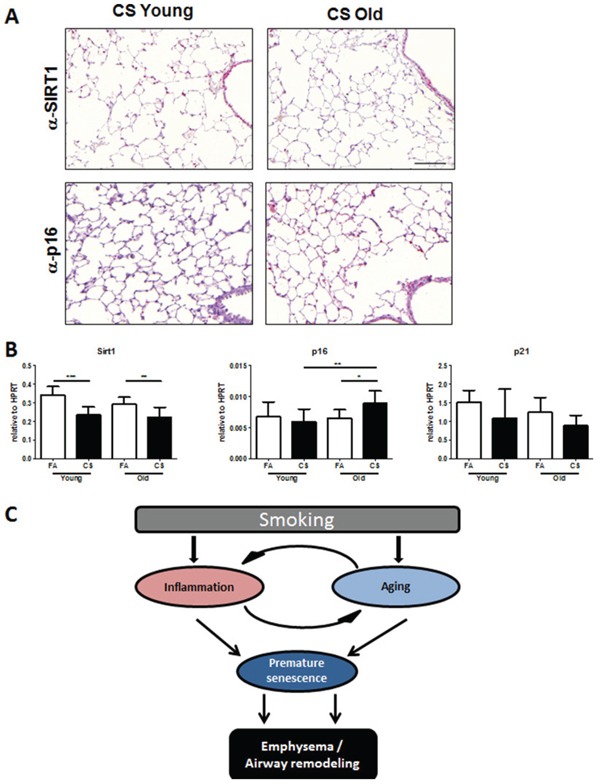
Senescence markers are elevated in the lungs of CS-exposed aged mice **A.** Representative micrographs of immunohistochemical stainings for anti-senescence marker SIRT1 and senescence marker p16 in lung sections from CS-exposed young and old mice; scale bar 100 μm. **B.** Lung tissue mRNA expression of SIRT1 and senescence markers p16 and p21 was measured by qPCR. Data were combined from 2 independent experiments with *n* = 8 and are given as mean values ± SD; one-way ANOVA following Bonferroni post test with *p < 0.05, **p < 0.01, ***p < 0.001. **C.** Schematic of proposed mechanism for accelerated COPD development in aged mice.

Previous studies reported that cellular senescence is involved in emphysema development [19]. Because SIRT1 is an anti-senescence gene that protects against elastase-induced emphysema via reduction of premature cellular senescence in mice [20], we performed immunohistochemistry for SIRT1 and senescence marker p16 in lung tissue of CS-exposed mice. We observed higher staining for SIRT1 in the lungs of young CS-exposed animals, specifically in alveolar and airway epithelial cells (Fig. 6A). In aged mice, CS exposure led to a decrease in SIRT1 positive cells and to an elevated staining of p16 in alveolar epithelial cells (Fig. 6A). We further analyzed mRNA expression of SIRT1, p16 and p21 in lung tissue and found a significant upregulation of p16 in CS-exposed aged mice (Fig. 6B).

Thus, we suggest a contribution of elevated senescence as a potential mechanism for the accelerated COPD development in aged mice after CS exposure.

## DISCUSSION

Inflammation is a major player in COPD pathogenesis and is associated with disease progression. Recently, the development of COPD is also suggested to be influenced by premature senescence of lung resident cells [20], but the contribution of age-related changes of immune cell functions to accelerated COPD pathogenesis remains unclear. Our study aimed at investigating the role of increased inflammatory processes in the aging lung to pathophysiological changes after long-term CS exposure of mice for 3 months. We observed that lung inflammation (the presence of innate and adaptive immune cells, iBALT formation, BAL inflammation and cytokine expression) after CS exposure was accelerated in aged mice leading to increased lung compliance, emphysema development and small airway remodeling. In contrast, young mice did not show any development of COPD characteristics at this early stage of CS exposure, suggesting a role for age-related inflammatory changes in COPD pathogenesis.

Aging is associated with increased levels of oxidative stress, changes in antiaging molecules and telomere length, and cellular and immunosenescence amongst others [1]. Cell senescence, the cellular equivalent of aging, leads to irreversible growth arrest and subsequently halts tissue repair mechanisms. Abnormal senescence, which induces premature aging, is proposed to be involved in the pathogenic mechanisms of COPD [11]. In line with this, telomerase RNA-null (*mTR^−/−^*) mice with short telomeres were more susceptible to CS-induced emphysema development, which was attributed to senescence of lung resident cells [15]. Increased senescence in COPD was already described for the lungs of smokers with emphysema, characterized by overexpression of the cellular senescence marker p16 and telomere shortening in alveolar type II (ATII) and endothelial cells [21]. CS also induces senescence in alveolar epithelial cells *in vitro* [22]. Furthermore, deficiency in anti-senescence protein SMP30 in mice promoted airspace enlargement after CS exposure for 8 weeks [14].

However, senescence also causes inflammation as shown by the increased production of various inflammatory cytokines by senescent cells [19]. In immune cells, senescence is strongly associated with persistently activated innate immunity and with possibly uncontrolled adaptive immunity as suggested by changes in neutrophils, macrophages and Tregs amongst others [9]. Neutrophil levels do not increase with age, but their activity is altered leading to impaired killing capacity, slower chemotaxis and enhanced production of reactive oxygen species. The effects on adaptive immune cells include a reduction in naïve T cells, expansion of memory cells, Tregs, CD8^+^ and CD4^+^ cells, and a decline in B cell lymphopoiesis [2]. Recently, Th17 cells were found to be increased in older individuals, indicative of a disturbed Th17/Treg balance during aging [23]. In the lungs of aging mice, an upregulation of inflammatory genes and elevated numbers of CD4^+^, CD8^+^, B cells and macrophages were observed [10]. Using senescent DBA/2 mice as a model of the aging lung, Calvi *et al*. demonstrated increased oxidative stress, cell death and elastase activation, followed by inflammatory cell infiltration, immune complex deposition and the onset of airspace enlargement [24].

The chronic CS-induced inflammation of the lung is mainly attributed to the progressive activation of immune cells over time, which is characterized by increasing infiltration of the airways by innate and adaptive immune cells [7]. Recently, we revealed that during the chronic phase starting from 1 month of CS exposure, lymphocytes start to infiltrate the lung and form ectopic LFs, so-called iBALT structures [6]. Further studies have confirmed elevated B cell numbers in the mucosa of large airways in COPD patients compared to controls [25], and the number of LFs present in the lung also increased with disease severity [26]. How adaptive immune cells organized into LFs contribute to disease pathogenesis, however, has remained unclear. We demonstrated that B cells organized into iBALT structures were involved in inducing and maintaining a severe inflammatory response after CS exposure, causing subsequent emphysema development by regulation of macrophage accumulation and macrophage-derived MMP12 production [6]. Here, we also observed increased tissue inflammation, iBALT formation, MMP12 expression and emphysema development in old mice exposed to CS for 3 months. This was related to increases in T cell subsets, specifically Th17 and Th2. In previous studies, Th17 cells and IL-17 were found to be involved in the development and regulation of tertiary lymphoid follicles such as iBALT [27].

Both CD4^+^ and CD8^+^ T cells have been described to play an important role in COPD pathogenesis. Motz *et al*. showed that chronic CS exposure led to the generation of pathogenic T cells capable of inducing COPD-like disease in *Rag2^−/−^* mice [28]. Shan *et al*. demonstrated that CS-induced Th17 inflammation and IL-17A secretion contributed to human and experimental emphysema development [29]. Interestingly, the overexpression of IL-17A in lung epithelial cells resulted in spontaneous inflammation in aging mice and was associated with increases in MMP9 and TIMP1 [30]. Moreover, CS-exposed *IL-17RA^−/−^* mice failed to induce CCL2 (MCP1) and MMP12 and did not develop emphysema after 6 months of exposure [31].

A possible involvement of Th2 cells specifically via the signature cytokine IL-13 in COPD development has only been demonstrated in one animal study. Zheng *et al*. showed that IL-13, classically regarded as a critical cytokine in asthma pathogenesis, caused emphysema development and inflammation, when overexpressed in lung epithelial cells [17]. This also led to induction of various cathepsins and MMPs, including MMP9 and MMP12. Furthermore, in the plasma of smokers or COPD patients, an increased IL-13 concentration was reported to be inversely correlated with the percentage of DFCO and FEV1 [32]. Most importantly, CD4^+^ cells in BAL fluid of COPD patients expressed significantly higher amounts of IL-13 than smokers and healthy controls [33].

Recently, two major studies have addressed the question of accelerated emphysema/COPD development in aging mice. The already mentioned work by Alder *et al*. identified telomere length as a determinant of CS-induced emphysema susceptibility [15]. Based on results obtained from bone marrow transfer experiments, the authors concluded that telomere length in lung resident cells, and not in circulating immune cells, was determining the increased susceptibility. However, a sidestream CS model was used in this study, and lung tissue inflammation after 6 months of CS exposure only consisted of macrophages. We recently published a comprehensive study on the differences between mainstream and sidestream CS exposure in an acute setting. We observed a strong inflammatory response characterized by neutrophilic influx, increased cytokine secretion, proinflammatory gene expression, and upregulated GM-CSF production only in the mainstream model [5]. Exposing mice to mainstream CS for up to 6 months was subsequently associated with strong tissue inflammation, iBALT formation and MMP12 expression [6].

The study by Zhou *et al*. did not find differences in CS-induced COPD development between young and old mice using the same age and strain as in our study [8]. Long-term CS exposure of young and old mice for 6 months did not reveal an effect of aging on COPD development, specifically emphysema and small airway remodeling, and was associated with similar pathophysiological changes. However, the main difference between these two studies is the shorter CS exposure for 3 months in ours compared to 6 months in Zhou *et al*. Along this line, we demonstrated that CS exposure of young mice for 4 months induced emphysema [6]. Extended exposure for 6 months, however, did not increase the severity of emphysema, indicating that maybe a certain level of disease severity was already reached at an earlier time point. Here, we clearly demonstrate by using lung function methods supplemented with histological analyses an accelerated COPD development after 3 months of CS exposure in aged mice.

In this context, Jurk *et al*. described that low-level chronic inflammation induced telomere dysfunction and accelerated aging in *Nfkb1*^−/−^mice lacking repressive subunits of NF-κB [34]. Interestingly, this also led to infiltration of immune cells and spontaneous development of LF-like structures in the lungs of these animals. Thus, we speculate that the elevated baseline inflammation observed in the lungs of our aged mice increases the risk for CS-induced acceleration of inflammation leading to COPD development. Indeed, we further hypothesize that the enhanced inflammation following CS exposure, on top of that already observed in the lungs of aged animals, is driving increased levels of senescence and a resultant acceleration in COPD pathogenesis (Fig. 6C). In accordance with this, we observed a reduction of SIRT1 and increased p16 expression only in the lungs of aged CS-exposed mice.

Taken together, our findings support the aging hypothesis of disease development in COPD. In our current study, inflammaging indeed contributed to the accelerated pathogenesis of emphysema and airway remodeling via an increased chronic lung tissue inflammation and increased inflammatory mediators. This points to a contribution of senescence/aging-related changes in immune cell functions, with increased activation and a potential decline in regulation, to CS-induced COPD pathogenesis.

## MATERIALS AND METHODS

### Animals and maintenance

8 to 10 weeks (2 months) and 56 weeks (12 months) old pathogen-free female C57BL/6 mice were obtained from Charles River (Sulzfeld, Germany) and housed in rooms maintained at constant temperature and humidity with a 12 hour light cycle. Animals were allowed food and water *ad libitum*. All animal experiments were conducted under strict governmental and international guidelines and were approved by the local government for the administrative region of Upper Bavaria.

### Cigarette smoke (CS) exposure

Cigarette smoke (CS) was generated from 3R4F Research Cigarettes (Tobacco Research Institute, University of Kentucky, Lexington, KY). Mice were whole body exposed to active 100% mainstream CS of 500 mg/m^3^ total particulate matter (TPM) for 50 min twice per day for 3 months in a manner mimicking natural human smoking habits as previously described [5]. Briefly, the smoke was drawn into an exposure chamber via a membrane pump. Control mice were kept in a filtered air (FA) environment, but exposed to the same stress as CS-exposed animals. 24 h after the last CS exposure, mice were sacrificed.

The TPM level was monitored via gravimetric analysis of quartz fiber filters prior and after sampling air from the exposure chamber and measuring the total air volume. CO concentrations in the exposure chamber were constantly monitored by using a GCO 100 CO Meter (Greisinger Electronic, Regenstauf, Germany) and reached values of 288 ± 74 ppm. All mice tolerated CS-mediated CO concentrations without any sign of toxicity, with CO-Hb levels of 12.2 ± 2.4%. Experiments were performed with *n* = 8 animals per group and were repeated twice. All animals were subjected to lung function analysis. Afterwards, *n* = 4 animals per group were lavaged, the right lung was shock-frozen in liquid nitrogen and the left lung was fixed in paraformaldehyde (PFA; see below). The remaining *n* = 4 animals per group were used for FACS analysis of whole lung single-cell suspensions (see below).

### Lung function measurement

Pulmonary function in mice was measured using a flexiVent system (Scireq, Montréal, Canada). Mice were anesthetized with ketamine-xylazine, intubated for the analysis at 2 months or tracheostomized at the end of the experiment and connected to the flexiVent system. Mice were ventilated with a tidal volume of 10 ml/kg at a frequency of 150 breaths/min in order to reach a mean lung volume similar to that of spontaneous breathing. Testing of lung mechanical properties including dynamic lung compliance and resistance was carried out by a software-generated script that took four readings per animal.

### Preparation of bronchoalveolar lavage (BAL)

BAL fluid was obtained to perform total and differential cell counts for inflammatory cell recruitment of neutrophils, macrophages and lymphocytes. The lungs were lavaged by instilling the lungs with 4 × 0.5 ml aliquots of sterile PBS (Gibco, Life Technologies, Darmstadt, Germany). For cytospins, cells were spun down at 400 g and resuspended in RPMI-1640 medium containing 10% FCS (both from Gibco). Total cell counts were determined in a hemocytometer. Differential cell counts were performed using morphological criteria on May-Grünwald-Giemsa-stained cytospins (200 cells/sample). BAL fluid was used to evaluate cytokine secretion via multiplex analysis.

### Lung tissue processing

Lung tissue was either shock-frozen in liquid nitrogen to isolate mRNA for gene expression analysis or fixed at a constant pressure (20 cm fluid column) by intratracheal instillation of PBS buffered 6% paraformaldehyde (PFA) and embedded into paraffin for histological analysis of hematoxylin-eosin (HE)-stained slides and for immunohistochemistry.

For analysis of lymphocyte infiltration, single-cell suspensions of whole lung tissue were used. Lungs were perfused with sterile PBS via the right ventricle to clear leukocytes and erythrocytes from the pulmonary circulation. Lung homogenization was performed via enzymatic digestion and mechanical dissociation steps using a lung dissociation buffer and the gentleMACS Dissociator (both from Miltenyi Biotec, Bergisch Gladbach, Germany). After dissociation, samples were applied to a filter to remove any remaining larger particles from the single-cell suspensions.

### Flow cytometry analysis of whole lung lymphocyte infiltration

For flow cytometric analysis of single-cell suspensions, one part of the sample was used for staining of regulatory T cells with the Mouse Regulatory T Cell Staining Kit (eBioscience, San Diego, CA). Remaining lung cells were subjected to density gradient centrifugation using Pancoll (PAN Biotech, Aidenbach, Germany) to isolate mononuclear cells. Isolated cells were cultured over night in anti-CD3/anti-CD28-coated plates to perform intracellular cytokine staining. On the following day, cultivated cells were restimulated with leukocyte activation cocktail with Golgi Plug (BD Pharmingen) for 4 h. Afterwards, cells were stained with anti-CD4 antibody (eBioscience), fixed in 2% formaldehyde, permeabilized in 0.3% saponin buffer and stained with antibodies against IL-17A, IFNγ (both from eBioscience) and IL-4 (Biozol) to distinguish between different T helper cell subpopulations. Multicolor analysis of stained cells was conducted with a BD FACSCanto II flow cytometer (BD Biosciences, Heidelberg, Germany) and BD FACSDiva software.

### Quantitative morphometry

Design-based stereology was used to analyze sections using an Olympus BX51 light microscope equipped with a computer-assisted stereological toolbox (newCAST, Visiopharm, Hoersholm, Denmark) on HE- or Masson's Trichrome stained lung tissue slides as previously described [6]. Air space enlargement was assessed by quantifying mean linear chord length (MLI) on 30 fields of view per lung. Briefly, a line grid was superimposed on lung section images. Intercepts of lines with alveolar septa and points hitting air space were counted to calculate MLI applying the formula MLI = ΣP_air_ × L(p)/ΣI_septa_ × 0.5. P_air_ are the points of the grid hitting air spaces, L(p) is the line length per point, I_septa_ is the sum of intercepts of alveolar septa with grid lines.

Volume of inducible bronchus-associated lymphoid tissue (iBALT) and airway collagen normalized to the basal membrane was quantified on 50 fields of view per lung by counting points hitting iBALT (P_iBALT_) or airway collagen (P_collagen_) and intercepts of lines with airways (I_airway_). The volume was calculated by applying the formula V/S = ΣP_iBALT/collagen_ × L(p)/ΣI_airway_.

### Immunohistochemistry

For immunohistochemistry, lungs were fixed in paraformaldehyde and embedded into paraffin. After deparaffinizing in xylene and rehydrating in alcohol, the tissue was treated with 1.8% (v/v) H_2_O_2_ solution (Sigma-Aldrich, St. Louis, MO) to block endogenous peroxidase. Heat induced epitope retrieval was performed in HIER Citrate Buffer (pH 6.0, Zytomed Systems) in a Decloaking chamber (Biocare Medical, Concord, CA). To inhibit nonspecific binding of antibodies, tissue slides were treated with a rodent blocking antibody (Biocare Medical). After overnight incubation with primary antibodies against MMP12 (Abcam, Cambridge, UK), CD45R/B220 (BD Pharmingen), CD21 (Novus Biologicals, Littleton, CO), CD3 (Sigma Aldrich), p16 (Santa Cruz, CA) or SIRT1 (Millipore, Schwalbach, Germany) tissue slides were incubated with an alkaline phosphatase-labeled secondary antibody (Biocare Medical). Signals were amplified by adding chromogen substrate Vulcan fast red (Biocare Medical). Slides were counterstained with hematoxylin (Sigma-Aldrich) and dehydrated in xylene. Afterwards, coverslips were mounted.

### Quantitative real time RT-PCR

Total RNA from lung tissue homogenate was isolated using a peqGOLD Total RNA Kit (Peqlab, Erlangen, Germany) according to the manufacturer's instructions. cDNA was synthesized using Random Hexamers and MuLV Reverse Transcriptase (Applied Biosystems, Darmstadt, Germany). mRNA expression of target genes KC (CXCL1), MCP1 (CCL2), MIP1α (CCL3), IL-6, TNF-α, MMP12, TIMP1, F4/80, CXCL13, IFNγ, Foxp3, Rorγt, Gata3, IL-13, SIRT1, p16 and p21 in comparison to housekeeping control hypoxanthine-guanine phosphoribosyltransferase (HPRT)-1 was determined using Platinum SYBR Green qPCR SuperMix (Applied Biosystems) on a StepOnePlus™ 96 well Real-Time PCR System (Applied Biosystems, Carlsbad, CA). Primers used are listed in Table 1. Relative transcript expression of a gene is given as 2^−ΔCT^ (ΔCt = Ct_target_ – Ct_reference_), relative changes compared to control are 2^−ΔΔCt^ values (ΔΔCt = ΔCt_treated_– ΔCt_control_). Primers were generated using Primer-BLAST software.

**Table 1:** Primers used for qPCR of lung tissue

Gene	Forward-Primer	Reverse-Primer
HPRT-1	CCT AAG ATG AGC GCA AGT TGA A	CCA CAG GAC TAG AAC ACC TGC TAA
KC (CXCL1)	CCG AAG TCA TAG CCA CAC	GTG CCA TCA GAG CAG TCT
MCP1 (CCL2)	CTT CTG GGC CTG CTG TTC A	CCA GCC TAC TCA TTG GGA TCA
MIP1α	CAC CAT ATG GCT CGG ACA CC	TCA GGA AAA TGA CAC CTG GCT
IL-6	GTT CTC TGG GAA ATC GTG GA	TGT ACT CCA GGT AGC TAT GG
TNF-α	CAC CAC GCT CTT CTG TCT	GGC TAC AGG CTT GTC ACT C
MMP12	TGT ACC CCA CCT ACA GAT ACC TTA	CCA TAG AGG GAC TGA ATG TTA CGT
TIMP1	CAC TGA TAG CTT CCA GTA AGG CC	CTT ATG ACC AGG TCC GAG TTG C
F4/80	CTC TGT GGT CCC ACC TTC AT	GAT GGC CAA GGA TCT GAA AA
CXCL13	TCT CTC CAG GCC AGG CAT TTC T	ACC ATT TGG CAC GAC GAT TCA C
IFNγ	TTC AGA GCT GCA GTG ACC	GAA GCA CCA GGT GTC AAG
Foxp3	AGA GCC CTC ACA ACC AGC TA	CCA GAT GTT GTG GGT GAG TG
Rorγt	CAA GTC ATC TGG GAT CCA CTA C	TGC AGG AGT AGG CCA CAT TAC A
Gata3	GTC ATC CCT GAG CCA CAT CT	TAG AAG GGG TCG GAG GAA CT
IL-13	TAC GGT CTC CAG CCT CCC CG	GGC CGT GGC GAA ACA GTT GC
SIRT1	CCA TTA ATG AGG AAA GCA ATA GGC	AAT ACA AGG CTA ACA CCT TGG G
p16	TCG TGA ACA TGT TGT TGA GG C	CTA CGT GAA CGT TGC CCA TC
p21	CGG TGT CAG AGT CTA GGG GA	AGA GAC AAC GGC ACA CTT TG

### Multiplex cytokine analysis

Concentrations of secreted cytokines and chemokines MCP1 (CCL2), KC (CXCL1), MIP-1α (CCL3), IL-6 and TNF-α in BAL were determined using a magnetic bead-based MILLIPLEX *MAG* multiplex assay (Millipore) and analyzed on a Luminex^100^ (BIO-RAD, Munich, Germany). For this assay, BAL fluid was concentrated (10×) by ultrafiltration in Amicon Ultra-0.5 centrifugal filter devices (Millipore).

### Flow cytometry analysis of splenocytes

Spleens were harvested from old and young mice, homogenized by mechanical dissociation using the gentleMACS Dissociator (Miltenyi Biotec) and filtered to obtain a single cell suspension. Total splenocytes were blocked with anti-mouse CD16/CD32 before staining with anti-mouse CD4, CD44, CD69 and CD62L (all from eBioscience). Stained cells were analyzed on a BD FACSCanto II flow cytometer (BD Biosciences) running BD FACSDiva software.

### Statistics

Results are given as mean values ± SD. One-way ANOVA following Bonferroni post test was used for all studies with more than two groups. Analyses were conducted using GraphPad Prism 6 software (GraphPad Software, La Jolla, USA).
